# Supplementary material for: Direct in-situ insights into the asymmetric surface reconstruction of rutile TiO2 (110)
Source: Nat Commun. 2024 Feb 22;15:1616. doi: 10.1038/s41467-024-46011-6 (PMC10883989; doi:10.1038/s41467-024-46011-6)
Supplement: Supplementary file 1 — Supplementary Information [file 41467_2024_46011_MOESM1_ESM.pdf]

Supplementary Information

Direct in-situ insights into the asymmetric surface reconstruction of rutile TiO_2 (110)

Wentao Yuan,[1,2,5] Bingwei Chen,[1,5] Zhong-Kang Han,[1,3] Ruiyang You,[1] Ying Jiang,[1] Rui Qi,[1] Guanxing Li,[1] Hanglong Wu,[1] Maria Veronica Ganduglia-Pirovano,[4] Yong Wang[1*]*

[1] *Center of Electron Microscopy and State Key Laboratory of Silicon Materials, School of Materials Science and Engineering, Zhejiang University, Hangzhou, 310027, China*

[2] *Shanxi-Zheda Institute of Advanced Materials and Chemical Engineering, Taiyuan, 030000, China.*

[3] *Fritz Haber Institute of the Max Planck Society, Faradayweg 4-6, 14195 Berlin, Germany.*

[4] *Institute of Catalysis and Petrochemistry, ICP-CSIC, C/Marie Curie 2, 28049 Madrid, Spain.*

[5] *These authors contributed equally.*

Correspondence Email: yongwang@zju.edu.cn; hanzk@zju.edu.cn

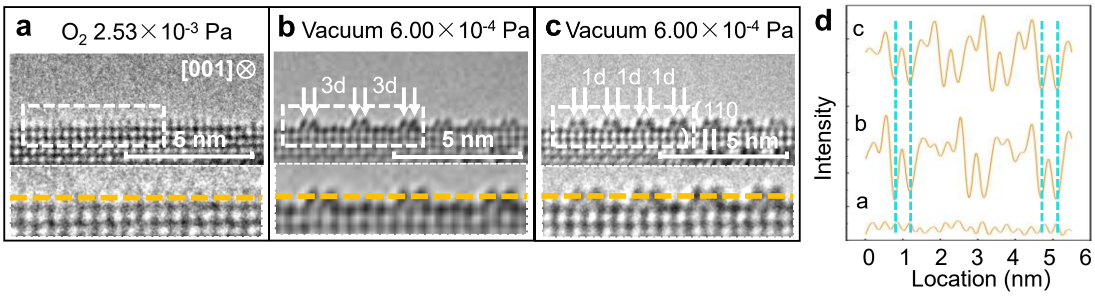

Supplementary Figure 1. The ETEM images of the (1×2) reconstructed rutile TiO_2 (110) surface. a-c Sequential in situ ETEM images of TiO_2 (110) surface at 700 °C under low oxygen pressure of **a** 2.53×10^{-3} Pa and **b,c** 6.00×10^{-4} Pa. The enlarged images of the dotted rectangles are shown in the lower panels of **a−c**, respectively. **d** Intensity profiles along the orange dashed lines in the lower panels of **a−c**. The dash lines are acquired from the reconstructed layer.

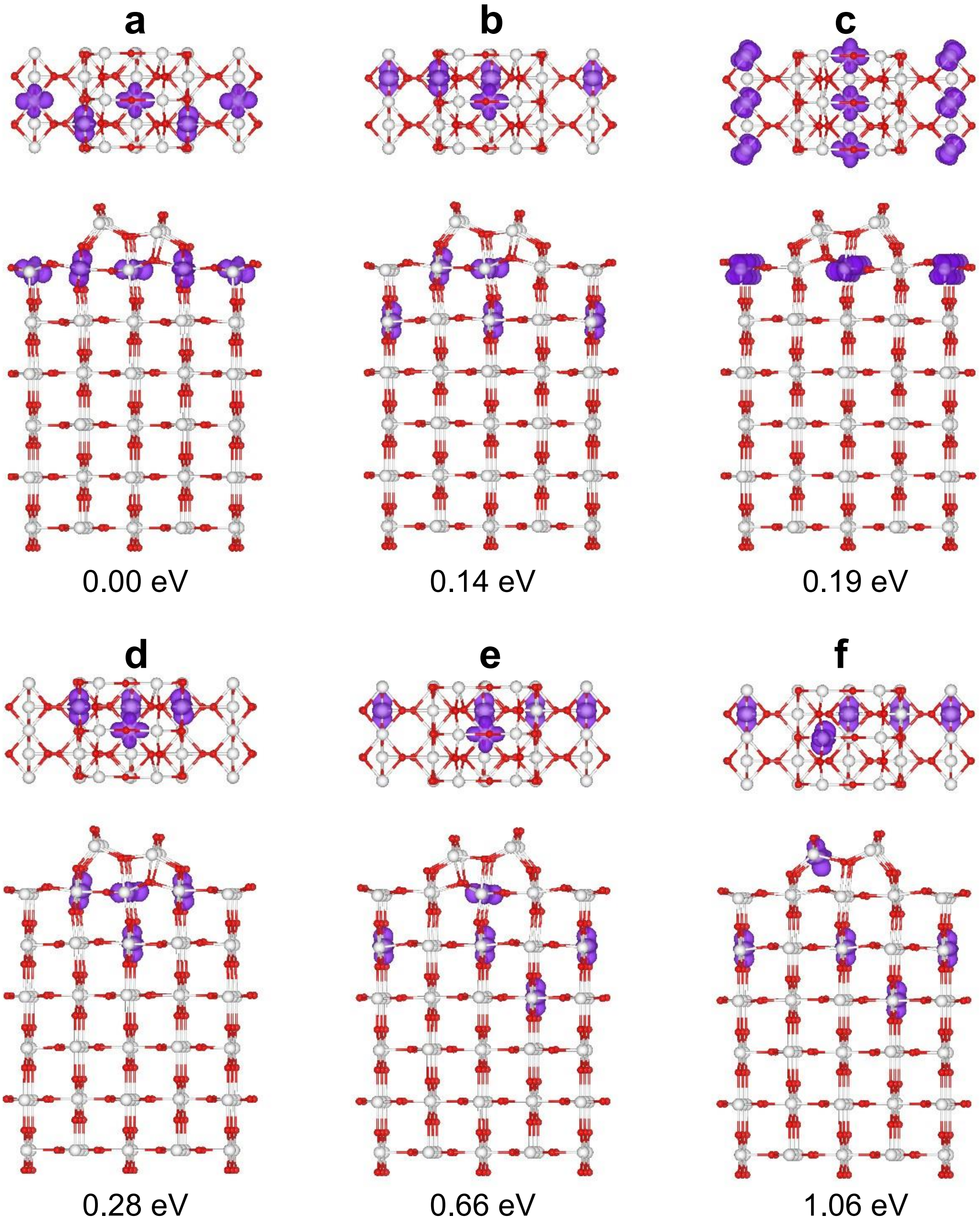

a
b
c
0.00 eV
0.14 eV
0.19 eV
d
e
f
0.28 eV
0.66 eV
1.06 eV

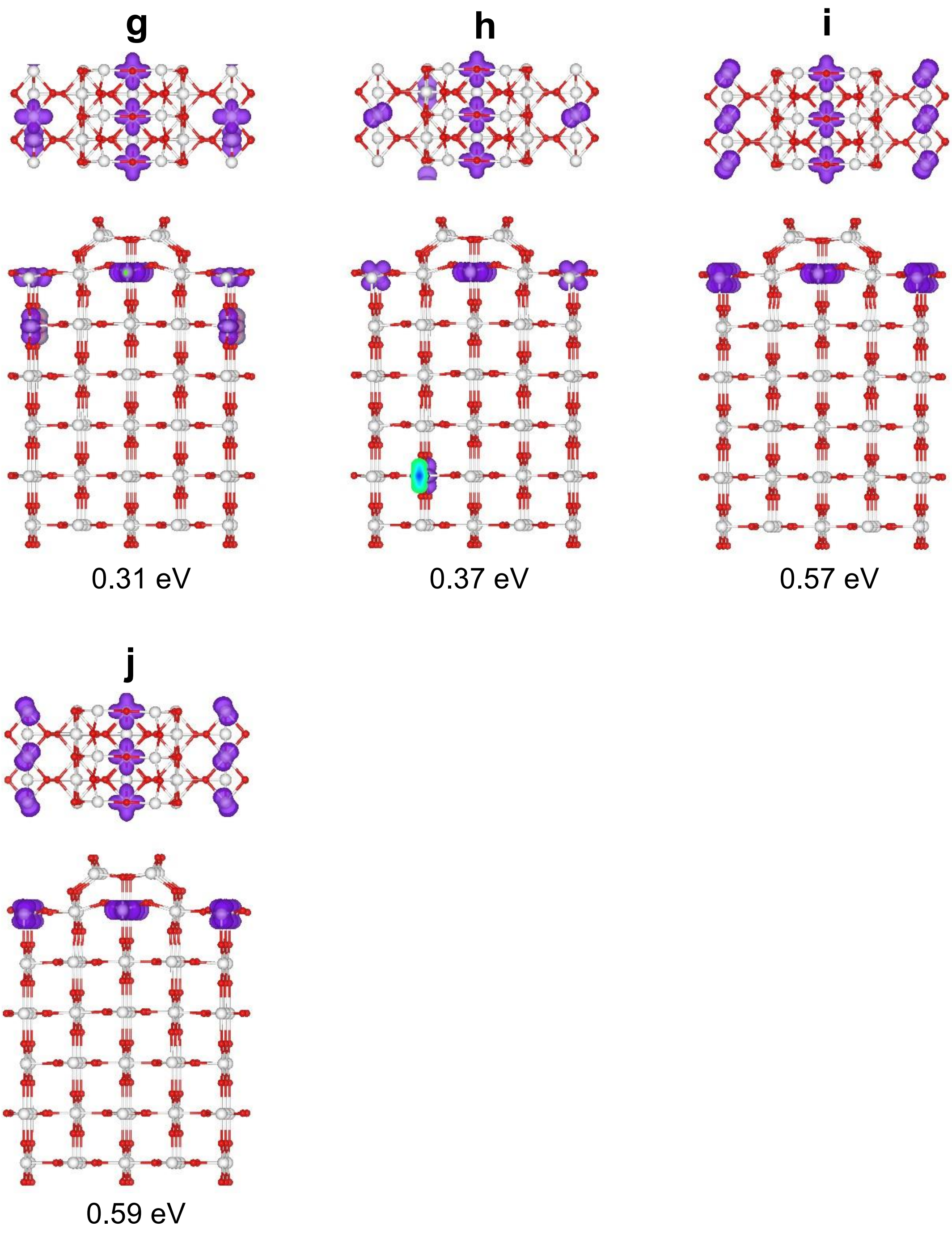

Supplementary Figure 2. Multiple distributions of the small polarons/Ti^{3+} ions in the near-surface of the Ti_2O_3-(1×2) reconstructions. a-f Asymmetric and **g-j** symmetric Ti_2O_3-(1×2) reconstructions, together with the spin charge density distribution. The titanium and oxygen atoms are represented by white and red balls, respectively. The energy values are relative to the most stable configuration.

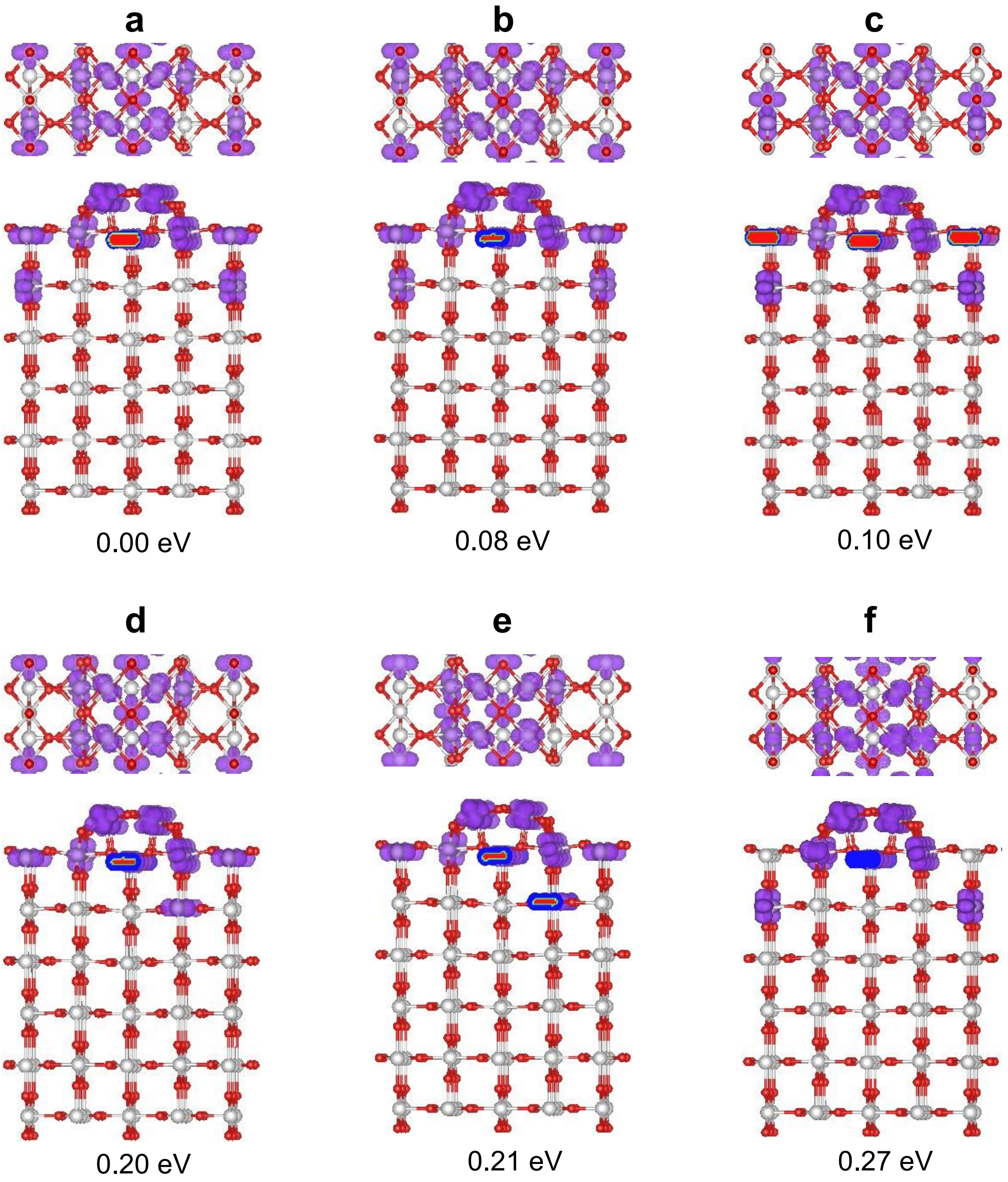

Supplementary Figure 3. Multiple distributions of the polarons in the near-surface of the Ti_2O-(1×2) reconstruction. The titanium and oxygen atoms are represented by white and red balls, respectively. The energy values are relative to the most stable configuration.

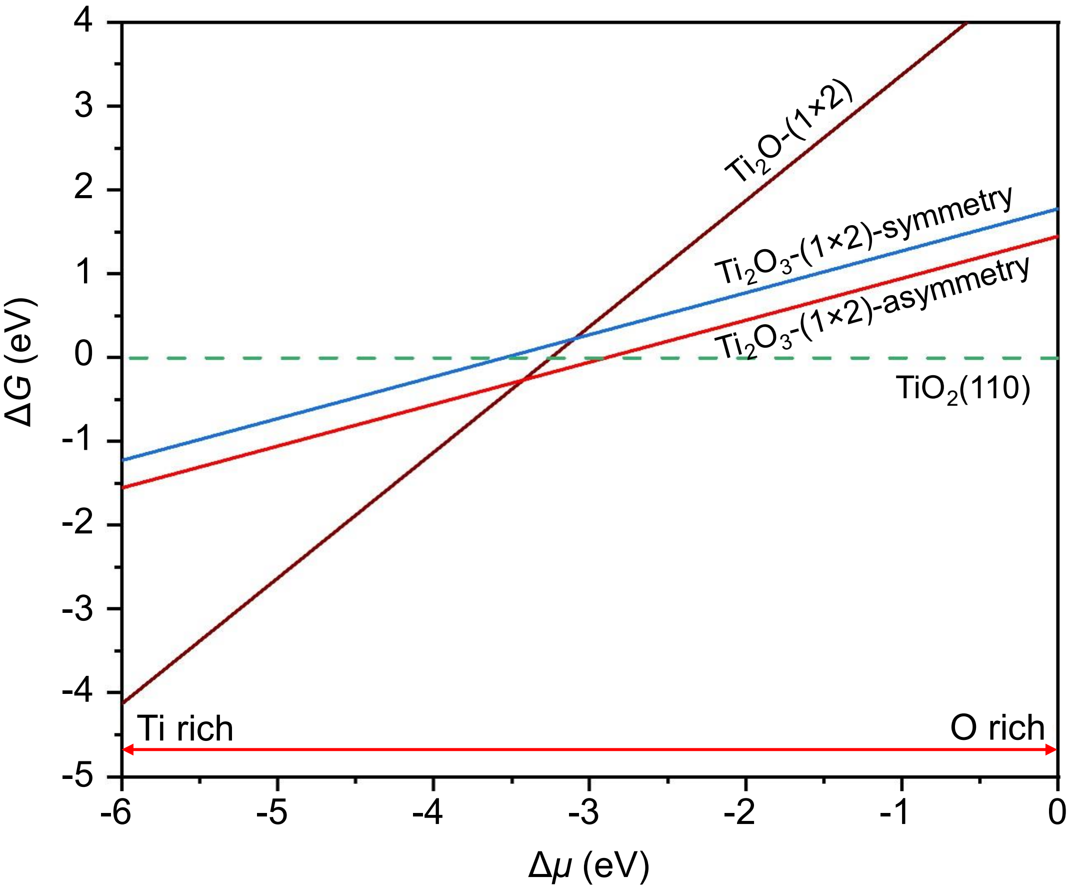

Supplementary Figure 4. Surface phase diagram of rutile TiO_2 (110): stability of different structures (Ti_2O-(1×2): brown; Ti_2O_3-(1×2)-symmetry: blue; Ti_2O_3-(1×2)-asymmetry: red) as a function of oxygen chemical potential $\Delta\mu$.

Supplementary Table 1. The formation energy of the asymmetric Ti_2O_3-(1×2) reconstruction relative to its symmetric counterpart at different temperatures, incorporating vibrational entropy correction.

Energy (eV)	0 K	300 K	600 K	900 K
	-0.31	-0.39	-0.47	-0.63

Supplementary Table 2. The formation energy of the asymmetric Ti_2O_3-(1×2) reconstruction relative to its symmetric counterpart for different computational setups.

Energy (eV)	2×1×1 *k*-points; 400 eV	2×1×1 *k*-points; 500 eV	4×2×1 *k*-points; 400 eV	4×2×1 *k*-points; 500 eV

U = 3.6	-0.31	-0.33	-0.32	-0.33
U = 4.1	-0.31	-0.33	-0.33	-0.34
U = 4.6	-0.32	-0.35	-0.33	-0.35

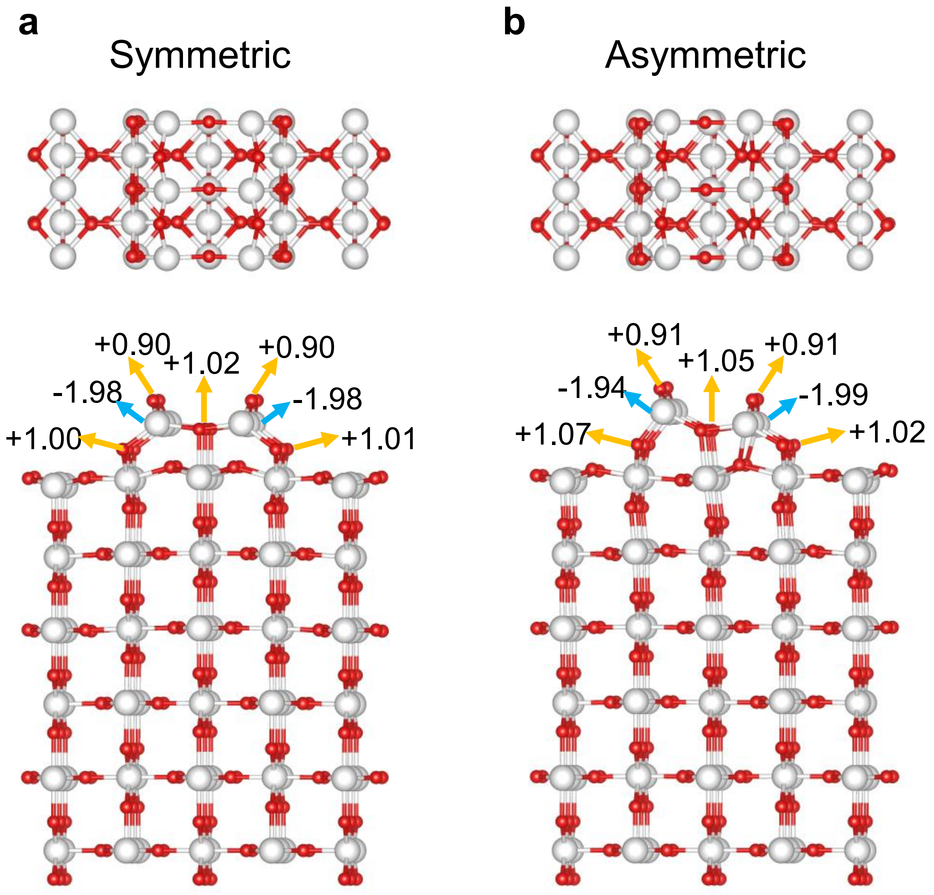

Supplementary Figure 5. The Bader charge distribution in the top layers of the Ti_2O_3-(1×2) reconstructions. The titanium and oxygen atoms are represented by white and red balls, respectively.

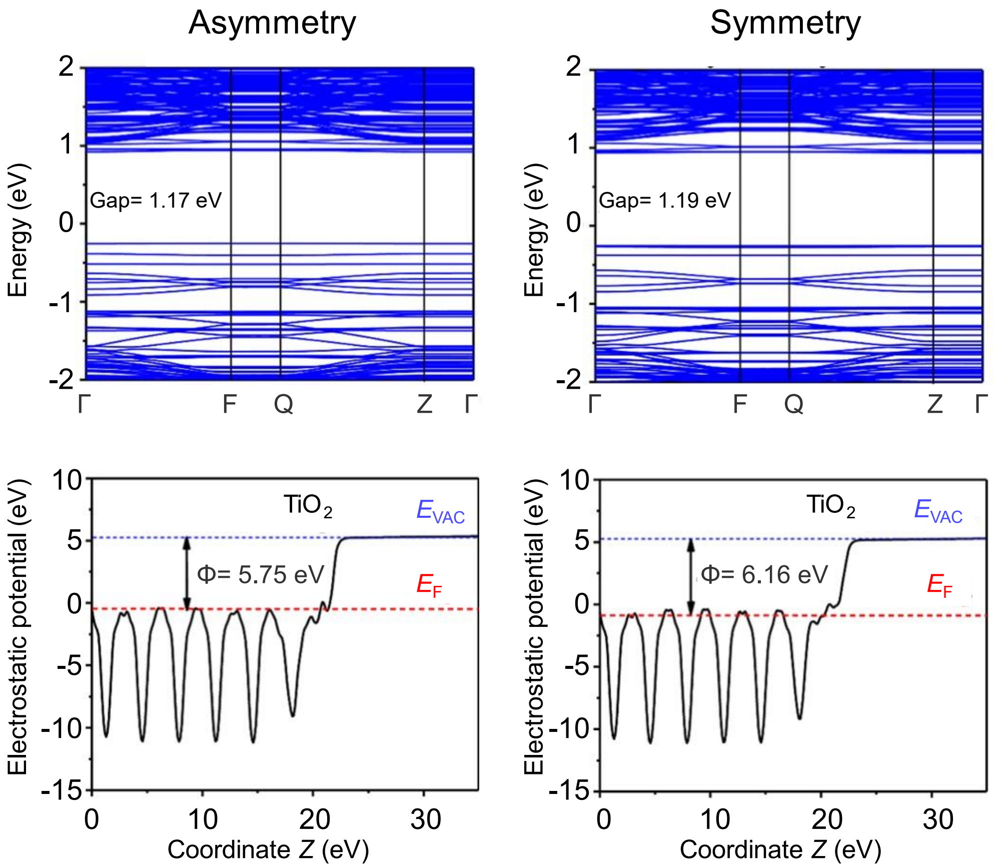

Supplementary Figure 6. Bandstructures and work functions of the most stable a, c asymmetric and b, d symmetric Ti_2O_3-(1×2) reconstructions.

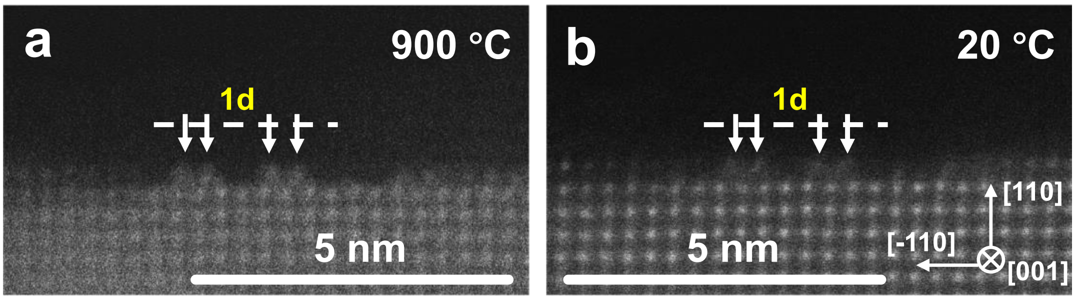

Supplementary Figure 7. The atomic-resolution HAADF STEM images of the (1×2) reconstructed rutile TiO_2 (110) surface. a-b Sequential in situ HADDF images of TiO_2 (110) surface at 900 °C (a) and 20 °C (b) (with “d” denoting the periodicity of the 1×1 bulk-terminated surface). These images are acquired *in situ* in a vacuum (≈10^{-5} Pa).
